# Functional Brain Connectivity Differences Between Different ADHD Presentations: Impaired Functional Segregation in ADHD-Combined Presentation but not in ADHD-Inattentive Presentation

**DOI:** 10.18869/nirp.bcn.8.4.267

**Published:** 2017

**Authors:** Amir Hossein Ghaderi, Mohammad Ali Nazari, Hassan Shahrokhi, Amir Hossein Darooneh

**Affiliations:** 1. Cognitive Neuroscience Laboratory, Department of Psychology, University of Tabriz, Tabriz, Iran.; 2. Research Center of Psychiatry and Behavioral Sciences, Tabriz University of Medical Sciences, Tabriz, Iran.; 3. Department of Physics, Faculty of Sciences, University of Zanjan, Zanjan, Iran.

**Keywords:** ADHD presentations, Graph theory, Brain segregation, Brain integration, EEG

## Abstract

**Introduction:**

Contrary to Diagnostic and Statistical Manual of Mental Disorders (DSM-5), fifth edition, some studies indicate that ADHD-inattentive presentation (ADHD-I) is a distinct diagnostic disorder and not an ADHD presentation.

**Methods:**

In this study, 12 ADHD-combined presentation (ADHD-C), 10 ADHD-I, and 13 controls were enrolled and their resting state EEG recorded. Following this, a graph theoretical analysis was performed and functional integration and segregation of brain network was calculated.

**Results:**

The results show that clustering coefficient of theta band was significantly different among three groups and significant differences were observed in theta global efficiency between controls and ADHD-C. Regarding the alpha band, a lower clustering coefficient was observed in control subjects. In the beta band, clustering coefficient was significantly different between the control and children with ADHD-C and also between ADHD-I and ADHD-C. The clustering coefficient, in the subjects with ADHD-C, demonstrated a rapid decline and was significantly lower than the subjects with ADHD-I and control.

**Conclusion:**

Decreased clustering, in high thresholds, may be associated with hyperactivity while increased segregation in low thresholds with inattentiveness. A different functional network occurs in the ADHD-C brain that is consistent with several studies that have reported ADHD-I as a distinct disorder.

## 1. Introduction

Attention-Deficit Hyperactivity Disorder (ADHD) in the Diagnostic and Statistical Manual of Mental Disorders-fifth edition (DSM-5) is characterized by a behavioral pattern present in multiple settings, including school and home (American Psychiatric Association, 2013). The prevalence of ADHD is 5% to 10% in school-aged children (Scahill & Schwab-Stone, 2000). Symptoms are divided into two categories of inattention and hyperactivity-impulsivity (American Psychiatric Association, 2013). Children with ADHD with a predominantly inattentive presentation (ADHD-I) have difficulties in focusing their attention while children with ADHD combined presentation (ADHD-C) have two categories of symptoms: inattention and hyperactivity-impulsivity.

Despite conventional categorization of ADHD presentations, some studies suggest that ADHD-I is a distinct diagnostic disorder and not a presentation of ADHD (Milich, 2001; Barkley, 2001). Barkley suggests that executive function deficit (EF-hypothesis) is not common in ADHD-I (Barkley, 1997) and this model is supported by other studies (Nigg, Blaskey, Huang-Pollock & Rappley, 2002; Lockwood, Marcotte, & Stern, 2001). However, the EF-hypothesis of ADHD-C is controversial (Geurts, Verte, Oosterlaan, Roeyers, & Sergeant, 2005; Willcutt, Doyle, Nigg, Faraone, & Pennington, 2005). Some evidence suggests that ADHD-C and ADHD-I presentations do not differ from one another and support the validity of the DSM classification of ADHD combined and inattentive presentations (Geurts et al., 2005; Willcutt et al., 2005; Mayes, Calhoun, Chase, Mink, & Stagg, 2008).

Neurophysiological studies have shown that pathophysiology of ADHD is associated with several abnormalities in cortical and subcortical regions (Cortese et al., 2012; Tian et al., 2008; Nazari et al., 2010) and various functional networks such as frontostriatal and frontotemporal circuits (Cortese et al., 2012; Tian et al., 2008; Nazari et al., 2010; Konrad & Eickhoff, 2010). Functional magnetic resistant imaging (fMRI) has shown hypo-activity of the frontoparietal (executive functions system) and ventral attentional networks in children with ADHD compared to the controls (Cortese et al., 2012). Many electroencephalography (EEG) studies have reported that ADHD is characterized by abnormal amplitude in brain waves; specifically in the theta and beta frequencies (Arns, Conners, & Kraemer, 2012; Loo & Barkley, 2005; Lazzaro et al., 1998; Clarke, Barry, McCarthy, & Selikowitz, 2001). There is sufficient evidence to support abnormal activity of alpha waves in children with ADHD (Hale et al., 2010; Nazari, Wallois, Aarabi, & Berquin, 2011). Despite various investigations into the neurophysiology of ADHD, no clear boundaries have been found between different ADHD presentations and many studies have failed to demonstrate significant differences (Barkley, Grodzinsky, & DuPaul, 1992; Buyck & Wiersema, 2014; Willcutt et al., 2012; Chhabildas, Pennington, & Willcutt, 2001). However, other studies have been shown clear differences between presentations (Nikolas, & Nigg, 2013; Vasserman, Bender, & MacAllister, 2013).

The functional and structural connectivity of complex networks are investigated using graph theoretical analysis. Several recent studies have demonstrated that graph theoretical analysis is a reliable approach to determine functional or structural brain connectivity abnormalities in various neurological, psychiatric, and cognitive disorders (Bullmore & Sporns, 2009; Rubinov & Sporns, 2010; Papo, Buldu, Boccaletti, & Bullmore, 2014; Stam et al., 2016; Finn er al., 2014). In 1998, Watts and Strogatz introduced the concept that “small world” networks are highly clustered, like regular networks, and have small shortest path similar to random graphs. It has been suggested that models with small world coupling exhibit increased synchronization and speed of signal-propagation (Watts & Strogatz, 1998).

Small world topology has been identified in many functional and structural brain connectivity networks (Bullmore & Sporns, 2009; Rubinov & Sporns, 2010; Papo et al., 2014; Stam et al., 2016), and altered small world network has been reported in several disorders such as schizophrenia (Liu et al., 2008; Yu et al., 2011), autism (Itahashi et al., 2014), Alzheimer (Stam, Jones, Nolte, Breakspear, & Scheltens, 2006), and ADHD (Wang et al., 2008; Xia, Foxe, Sroubek, Branch, & Li, 2014; Cao, Shu, Cao, Wang, & He, 2014; Liu, Chen, Lin, & Wang, 2014). Recent studies suggest that functional connectivity in EEG sub-bands is altered during ADHD (Liu, et al., 2014; Ahmadlou, Adeli, & Adeli, 2012; Liu, Lin, Chen, & Wang, 2014). Decreased global efficiency, increased shortest path length and local characteristics in ADHD have been confirmed in several studies (Liu et al., 2014; Cao et al., 2013). In addition, altered connectivity of orbitofrontal-temporal and frontal-amygdala networks has been demonstrated in subjects with inattention and hyperactivity in recent fMRI studies (Cocchi et al., 2012; Mills et al., 2012). However, the functional difference of complex brain networks in ADHD presentations has not been investigated yet.

The current study is the first study to identify a functional difference in ADHD presentations using graph theoretical analysis. EEG coherence in different frequency bands (delta, theta, alpha, and beta) among electrode pairs was used to determine edges and clustering coefficients, characteristic path lengths and the clustering coefficient for ADHD-I, ADHD-C, and control groups. Finally, the topological properties of the brain networks were demonstrated and the functional differences among three groups were characterized.

## 2. Methods

### 2.1. Participants

Thirty-five right-handed children aged between 7 and 11 years participated in the experiment 12 ADHD-C [8 boys, 4 girls; mean age: 8.42±1.78 y], 10 ADHD-I [6 boys, 4 girls; mean age: 8.60±1.42 y] and 13 controls [8 boys, 5 girls; mean age: 8.92±1.38 y]. Children with ADHD were all recruited from Hamrah Child and Adolescent Multidisciplinary Neuropsychiatric Center, Tabriz, Iran. None of the children in the study had ever been treated with methylphenidate or had a history of treatment with neuromodulation devices such as neurofeedback or transcranial direct current stimulation. Full diagnostic criteria for the ADHD combined and inattentive participants for ADHD-C and ADHD-I were implemented, respectively. The diagnosis was based on DSM-5 criteria.

For all participants, the child behavior checklist (Tehrani-Doost, Shahrivar, Pakbaz, Rezaie, & Ahmadi, 2011) was completed by the parents; also the Swanson, Nolan, and Pelham IV questionnaire (Hooshyari, Mohammadi, & Delavar, 2008) was filled out by both parents and teachers. Then, the diagnostic interview schedule for children was applied. Diagnosis of the participants was also investigated independently by a child psychiatrist and a psychologist both blinded to the findings and was included in the groups if and only if both clinicians agreed on the diagnosis. Furthermore, children with other confounding neuropsychiatric disorders were excluded from the subjects.

### 2.2. EEG data acquisition and processing

For EEG recording, we used a Mitsar® amplifier with 21 channels and WinEEG® software. EEG was sampled at 250 Hz with a filtered online 0.1–40 Hz band pass. EEG was recorded using a 19 channel Electrocap® according to the 10–20 international system. All electrodes were referenced to linked earlobes, and the ground was placed on AFZ. Electrode impedance was maintained below 10 kΩ.

EEG signals were recorded for 5 minutes in eye open condition and at least 90 seconds of artifact free signal was selected for processing. Visual signal selection and then automatic rejection (the amplitude threshold detection algorithm in Neuro-guide® software) was used for artifact rejection. Each epoch was also visually appraised by an independent expert. EEG data were processed offline by Neuro-guide software^1^ and the EEG coherence of 5 frequency bands; theta (4.5–7 Hz), alpha (7.5–12 Hz), beta1 (12–15 Hz), beta2 (15–17.5 Hz) and beta3 (18–25 Hz) among all electrode pairs (total 171 pairs×5 bands) were obtained.

## 2.3. Graph theoretical analysis

A 19×19 binary adjacent matrix was formed as a functional network for each participant. Previous findings have suggested that coherence can be used to characterize the pathophysiological changes in ADHD (Barry, 2002). Therefore, to identify the functional network between electrodes, the EEG spectral coherence between all electrode pairs was used (171 pairs in each frequency). Coherence was calculated by:

$${{\mathrm{Coh}}^{2}}_{\mathrm{ij}}(\omega )=\frac{E\left[{C_{ij}(\omega )|}^{2}\right]}{E\left[C_{\mathrm{ii}}\left(\omega \right)\right]E\left[C_{\mathrm{jj}}\left(\omega \right)\right]}$$

Where C(w) is the Fourier transform of cross correlation coefficients between two electrodes (Sanei & Chambers, 2007). Binary adjacency matrixes or undirected graphs were produced by applying a threshold to each element of the coherence matrixes. The networks were then investigated across a broad range of thresholds.

As a measure of functional segregation (Rubinov & Sporns, 2010), the clustering coefficient (C) was calculated for each participant. The clustering coefficient in a node *i* is obtained by counting the number of edges in the subgraph of *i* neighbors. Locally, the clustering coefficient is measured as the ratio between the number of actual edges and the maximum possible number of edges in the subgraph of neighbors of *i* (Boccaletti, Latora, Moreno, Chavez, & Hwang, 2006). Therefore, the clustering coefficient reflects the extent to which neighbors of a vertex are also neighbors of each other (Watts & Strogatz, 1998).

Functional integration in the brain is measured by the characteristic path length of the network (L) that is identified by averaging on all shortest paths. In a 19×19 binary adjacent matrix, there are 171 shortest paths between all pairs of nodes in the network and their average is the characteristic path length of the network (Rubinov & Sporns, 2010). Rapid integration of specialized information from distributed brain regions was best seen with a small characteristic path length (Rubinov & Sporns, 2010).

Clustering coefficient and global efficiency were calculated as a function of threshold using the open source Matlab toolbox^2^ (Rubinov & Sporns, 2010) and the results were compared among three groups. After wards, small world structure was investigated. Watts and Strogatz (1998) proposed a method by which C and L can be used for quantifying the small-world properties of a network. As indicated by Watts and Strogatz, a small-world network can be highly clustered as regular graphs. In addition, the small-world network has small characteristic path lengths consistent with random networks (Watts & Strogatz, 1998). In other words, small world graphs are simultaneously highly integrated and segregated (Rubinov & Sporns, 2010).

### 2.4. Statistical analysis

Statistical analysis was accomplished with IBM SPSS 19. Average clustering coefficient and average global efficiency among all electrodes in each group were compared by analysis of variance (ANOVA). To investigate differences between group pairs, a post hoc analysis (Tukey) was applied. In various thresholds of frequency bands, clustering coefficient and global efficiency were calculated and related graphs as a function of thresholds were determined. In each band, the R among these graphs were obtained by Matlab software.

## 3. Results

### 3. 1. Theta band (4–7 Hz)

**Figure 1:**
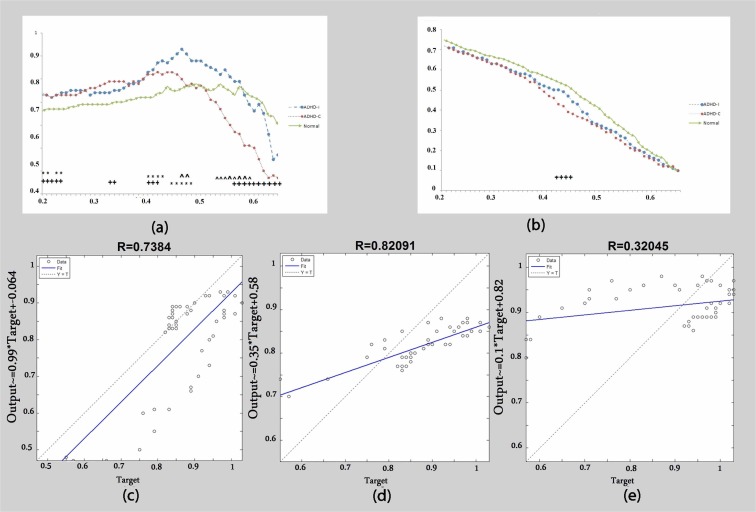
Theta band: (a) Clustering coefficient calculated as a function of threshold. (b) Global efficiency calculated as a function of threshold. (c) The correlation of clustering coefficient graphs between the ADHD-C group and ADHD-I group. (d) The correlation of clustering coefficient graphs between the control group and ADHD-I group. (e) The correlation of clustering coefficient graphs between the control group and ADHD-C group. + Significant difference between the control group and ADHD-C group. * Significant difference between the control group and t ADHD-I group. ^ Significant difference between the ADHD-C group and ADHD-I group. Big signs: P<0.01. Small signs: P<0.05.

Regarding the theta frequency band, the clustering coefficient was significantly different among three groups (Figure 1-a). In lower thresholds, both ADHD presentations displayed higher clustering values than the control group. However, in higher thresholds there was a rapid decline of clustering in the ADHD-C presentation and significant differences were observed between the ADHD-C and ADHD-I presentations. At high thresholds, clustering was significantly lower in ADHD-C than that in the control subjects.

Clustering coefficients as a function of different thresholds are shown in Figure 1-c. The correlation among three graphs was determined by calculation of R and a considerable correlation between control subjects and the ADHD-I group was observed (Figure 1-d and Table 1). However, the correlation between the ADHD-C and the control group was very weak (Figure 1-e and Table 1). Global efficiency was higher in the control group than either of the ADHD presentations and significant differences were observed between controls and ADHD-C (Figure 1-b).

**Table 1:** Correlation among the clustering graphs as functions of threshold.

**Band**	**ADHD-C and ADHD-I (R)**	**Normal and ADHD-I (R)**	**Normal and ADHD-C (R)**
Theta (4.5–7.5)	0.74	0.82	0.32
Alpha (8–12)	0.66	0.94	0.65
Beta 1 (12–15)	0.53	0.89	0.45
Beta 2 (15–18)	0.58	0.94	0.68
Beta 3 (18–25)	0.00	0.39	0.26

### 3. 2. Alpha band (8–12 Hz)

**Figure 2:**
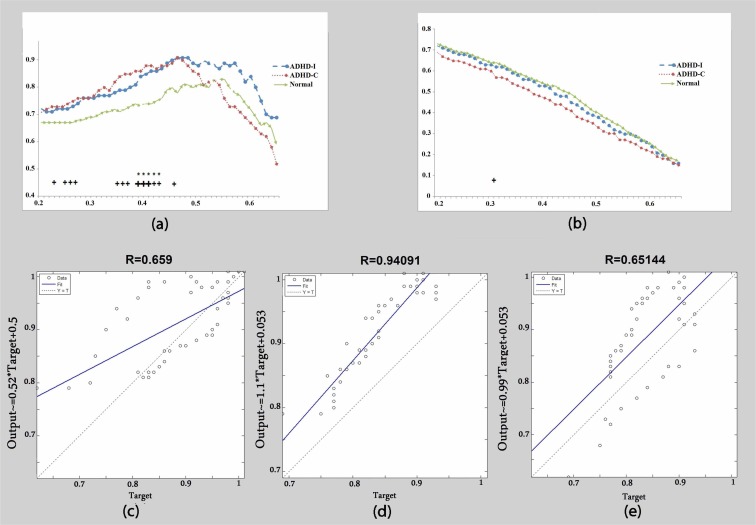
Alpha band: (a) Clustering coefficient calculated as a function of threshold. (b) Global efficiency calculated as a function of threshold. (c) The correlation of clustering coefficient graphs between the ADHD-C group and ADHD-I group. (d) The correlation of clustering coefficient graphs between the control group and ADHD-I group. (e) The correlation of clustering coefficient graphs between the control group and ADHD-C group. + Significant difference between the control group and ADHD-C group. * Significant difference between the control group and ADHD-I group. ^ Significant difference between the ADHD-C group and ADHD-I group. Big signs: P<0.01. Small signs: P<0.05.

In the alpha frequency band, a lower clustering coefficient was observed in control subjects compared to either of the ADHD presentations (T<0.5). In addition, a rapid decline in ADHD-C was observed in T>0.5 (Figure 2-a). The correlation of the clustering coefficient was considerable (R=0.94) as a function of thresholds between control and ADHD-I (Figure 2-d and Table 1). However, there was no significant correlation between controls and ADHD-C (Figure 2-e and Table 1). There was no significant difference between ADHD-I and ADHD-C (Figure 2-c and Table 1). Global efficiency in the control group showed a significant difference with ADHD-C at T=0.31, where lower values of global efficiency were observed particularly in ADHD-C presentation (Figure 2-b).

### 3. 3. Beta1 band (12–15 Hz)

**Figure 3:**
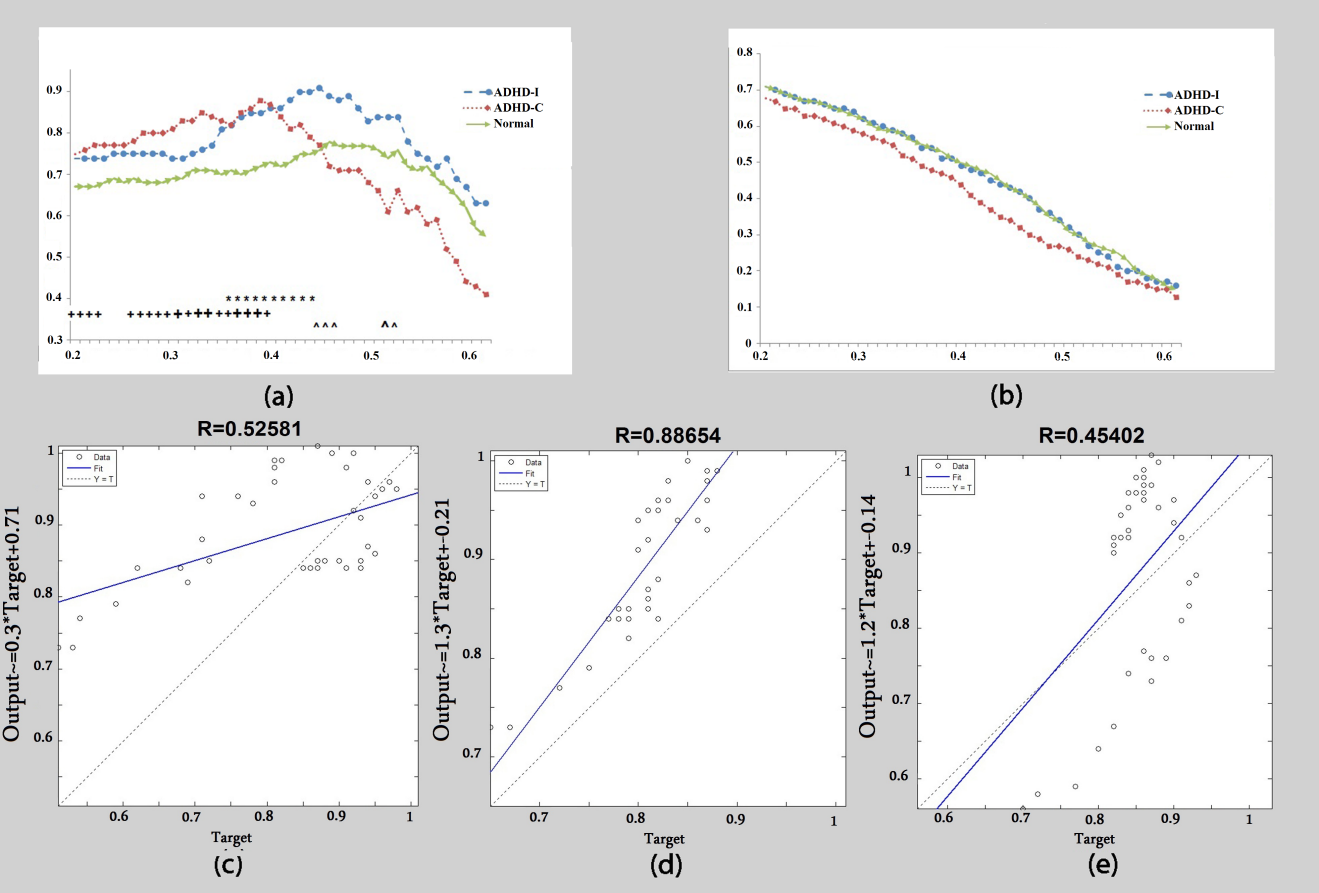
Beta1 band: (a) Clustering coefficient calculated as a function of threshold. (b) Global efficiency calculated as a function of threshold. (c) The correlation of clustering coefficient graphs between the ADHD-C group and ADHD-I group. (d) The correlation of clustering coefficient graphs between the control group and ADHD-I group. (e) The correlation of clustering coefficient graphs between the control group and ADHD-C group. + Significant difference between the control group and ADHD-C group. * Significant difference between the control group and ADHD-I group. ^ Significant difference between the ADHD-C group and ADHD-I group. Big signs: P<0.01. Small signs: P<0.05.

A significant difference in the beta1 frequency band was observed between the control group and children with ADHD-C in lower thresholds (T<0.4) and between ADHD-I and control subjects in 0.35<T<0.46. As in alpha and theta bands, a rapid decline in ADHD-C was observed in T>0.4 (Figure 3-a). The R between the clustering graphs as a function of threshold demonstrated a higher correlation between the control group and ADHD-I (R=0.89) than between the control group and ADHD-C (R=0.45) (Figures 3-d and 3-e). Furthermore, no remarkable correlation was observed between ADHD presentations (Figure 3-c). Both the control and ADHD-I groups demonstrated a higher global efficiency than ADHD-C group in a greater number of thresholds, but these differences were not significant (Figure 3-b).

### 3. 4. Beta2 band (15–18 Hz)

**Figure 4:**
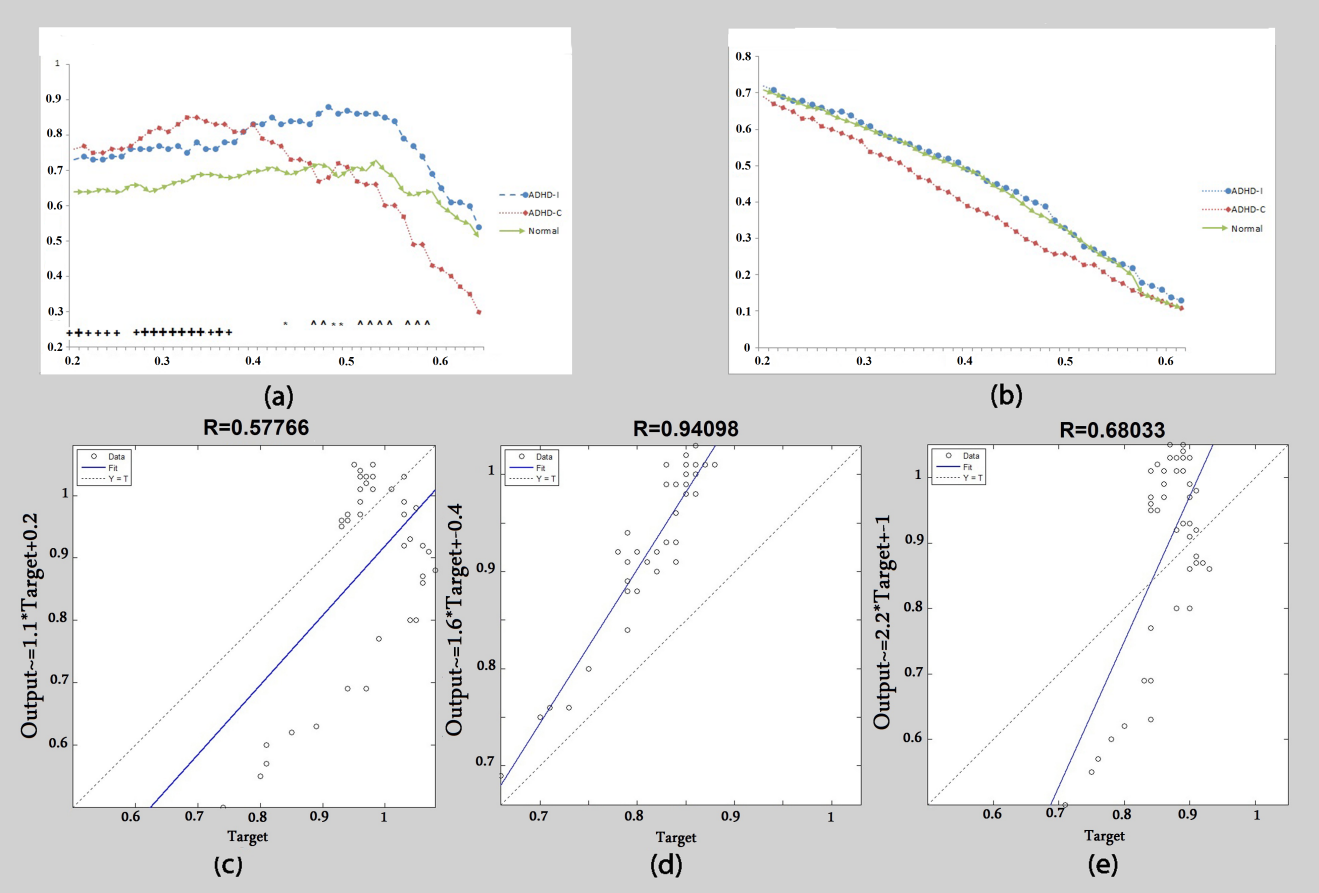
Beta2 band: (a) Clustering coefficient calculated as a function of threshold. (b) Global efficiency calculated as a function of threshold. (c) The correlation of clustering coefficient graphs between the ADHD-C group and ADHD-I group. (d) The correlation of clustering coefficient graphs between the control group and ADHD-I group. (e) The correlation of clustering coefficient graphs between the control group and ADHD-C group. + Significant difference between the control group and ADHD-C group. * Significant difference between the control group and ADHD-I group. ^ Significant difference between the ADHD-C group and ADHD-I group. Big signs: P<0.01. Small signs: P<0.05.

In the beta2 frequency band clustering coefficient, a significant difference was observed between the ADHD-C and control groups in T<0.38 while ADHD-C had a higher C than both ADHD-I and control groups (Figure 4-a). Regarding the middle thresholds (0.4<T<0.5), the ADHD-I group showed significantly higher values than the ADHD-C group and control subjects. At high thresholds (T>0.5), ADHD-I showed a significantly higher clustering coefficient than ADHD-C. ADHD-C displayed a rapid decline of the clustering coefficient graph at T>0.39 and there was no significant correlation between the clustering graph in ADHD-C and the control group (Figure 4-e). There was no correlation between ADHD-C and ADHD-I (Figure 4-c). However, as indicated in Figure 4-d, a significant correlation (R=0.94) was observed in the clustering coefficient graph as a function of threshold between ADHD-I and the control group.

### 3. 5. Beta3 band (18–25 Hz)

**Figure 5:**
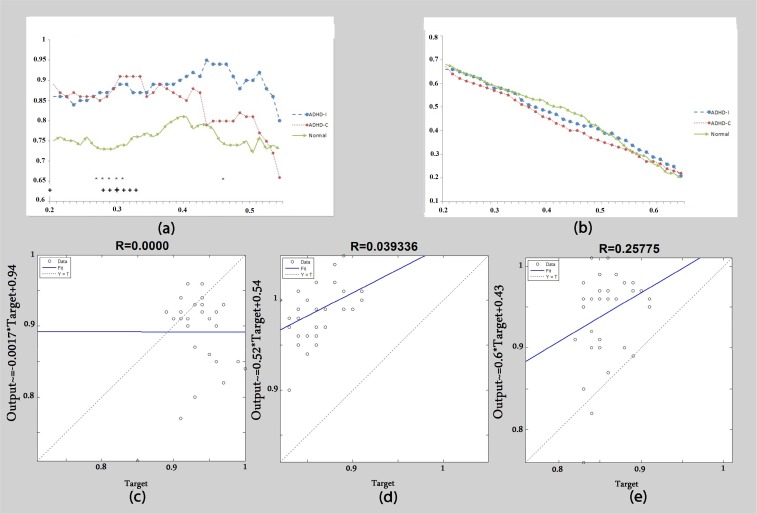
Beta 3 band: (a) Clustering coefficient calculated as a function of threshold. (b) Global efficiency calculated as a function of threshold. (c) The correlation of clustering coefficient graphs between the ADHD-C group and ADHD-I group. (d) The correlation of clustering coefficient graphs between the control group and ADHD-I group. (e) The correlation of clustering coefficient graphs between the control group and ADHD-C group. + Significant difference between the control group and ADHD-C group. * Significant difference between the control group and ADHD-I group. ^ Significant difference between the ADHD-C group and ADHD-I group. Big signs: P<0.01. Small signs: P<0.05.

In the beta3 frequency band, the clustering coefficients of ADHD-I and ADHD-C were significantly different from the control group at lower thresholds. In addition, there was no significant correlation between the clustering coefficient graphs (Figure 5-c, 5-d, and 5-e). Despite some correlations in other band frequencies, a significant correlation was not observed between the ADHD-I and control groups in the clustering coefficient graph as a function of threshold. The global efficacy was also not significantly different among three studied groups (Figure 5-b).

## 4. Discussion

**Figure 6:**
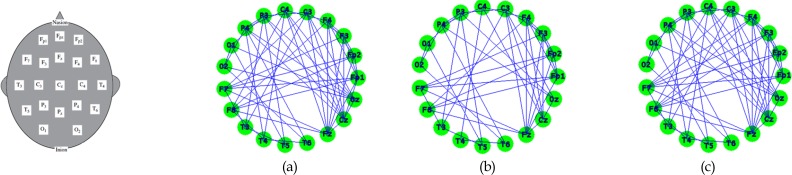
Theta band: Circular graph in T=31. (a) Control group. (b) ADHD-C group. (c) ADHD-I group.

**Figure 7:**
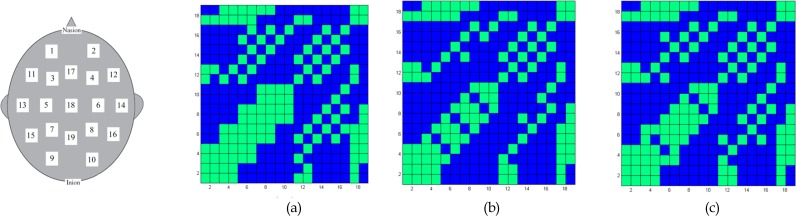
Alpha band: binary connectivity graph in T=30. (a) Control group. (b) ADHD-C group. (c) ADHD-I group Blue: disconnected nodes; Green: connected nodes.

The current study is the first to investigate functional connectivity differences between two ADHD presentations (ADHD-I and ADHD-C) and control subjects. We demonstrated that the functional connectivity in ADHD presentations is different from control subjects (Figure 6 and 7). However, children with ADHD-C exhibited more significant differences from control subjects compared to children with ADHD-I (Table 1). Significant differences were also observed between the ADHD-I and ADHD-C groups.

Several studies suggest that stimulus assessment (Brickman et al., 2005; Sammer et al., 2007) and Long-Term Potentiation (LTP) (Sammer et al., 2007; Larson, Wong, & Lynch, 1986) are related to EEG theta activity. Likewise, many conventional studies report that theta hyperactivity is observed in ADHD (Arns, et al., 2012; Loo & Barkley, 2005; Lazzaro et al., 1998; Clarke, Barry, McCarthy & Selikowitz, 2001). Previous studies have also shown that children with ADHD exhibit elevated intra hemispheric coherence in the theta band. These studies have demonstrated that children with ADHD-C have greater intra hemispheric theta coherence than children with ADHD-I (Barry, 2002).

Evidently a greater theta coherence reported in children with ADHD may be associated with hyperactivity (Barry, 2002; Clarke et al., 2008). In agreement with these previous findings, the present study used complex network analysis to expose different functional brain networks in children with ADHD. Specifically, at higher thresholds, a rapid decline of the clustering coefficient was observed in the ADHD-C group. Furthermore, the clustering coefficient for this group was significantly lower than the ADHD-I group and control subjects.

Alpha waves have also been investigated in several ADHD studies (Hale et al., 2010; Nazari et al., 2011). These studies have demonstrated that children with ADHD had reduced intra hemispheric coherences in the alpha bands. At longer inter electrode distances, ADHD children had lower intra hemispheric alpha coherence than controls (Barry, 2002). In a separate alpha analysis, increased coherence in the lower alpha (8 Hz) and decreased coherence in the upper alpha (10–11 Hz) band was seen in the ADHD group (Murias, Swanson, & Srinivasan, 2006). Alpha coherence has been shown to be stronger during observation of movement than during pauses in activity (Van der Helden, Van Schie, & Rombouts, 2010). However, reduced alpha 1 coherence, increased alpha 2, and beta coherence have been observed in anticipatory actions (Silva et al., 2012). Our results show that the alpha clustering coefficient in ADHD presentations is higher than normal. However, at higher thresholds (T>0.45), a rapid decline was observed in ADHD-C but not in ADHD-I.

A reduced beta power has been repeatedly reported in ADHD children (Arns et al., 2012). In the present study, children with ADHD-C were shown to have increased beta1 and beta2 clustering coefficients at low thresholds and a significant difference was observed between ADHD-C and control subjects (Figure 3-a). At higher thresholds, as in theta and alpha bands, children with ADHD-C show a rapid decline in the clustering coefficient and significant differences were observed between ADHD-I and ADHD-C groups. There was no significant change in global efficiency (Figure 3-b).

The presence of clusters or modules in functional networks denotes a segregated neural processing (Rubinov & Sporns, 2010). The ability to rapidly combine specialized information from distributed brain regions requires functional integration of the brain and is correlated by characteristic shortest path or inversely by global efficiency (Rubinov & Sporns, 2010). In low thresholds, the functional brain network in ADHD presentations was significantly different from that in control subjects (in theta, alpha, beta1 and beta2 bands). A high clustering coefficient, in ADHD presentations, was associated with increased local modularity in ADHD brain. This finding is consistent with previous studies (Lin et al., 2013; Wang, Lin, & Wu, 2014) and may be related to inattention.

The small world brain function has been demonstrated in previous studies (Bullmore & Sporns, 2009; Rubinov & Sporns, 2010; Papo et al., 2014; Stam et al., 2016). A small-world network is highly clustered with low characteristic path (or high global efficiency). Increased theta clustering coefficient and decreased theta global efficiency in ADHD-C compared to the control group demonstrates a shift toward regular networks and this finding is in agreement with a previous fMRI study (Wang et al., 2008). A significant difference in the clustering coefficient (T>0.49) between the ADHD-I group and the ADHD-C group is in agreement with previous studies indicating that abnormal activity in the theta band is correlated to hyperactivity (Barry, 2002; Clarke et al., 2008). This is an important result, because there is no significant difference between the ADHD-I group and control subjects at these thresholds.

Overall, an impaired segregation in higher thresholds was observed in children with ADHD-C (in theta, alpha, beta1, and beta2 bands). This new finding indicates a brain functional change in the ADHD-C presentation that was completely different from the ADHD-I presentation. This suggests that various neuronal activities may be involved in different ADHD-presentations. The current finding is consistent with previous studies that introduce ADHD-I as a distinct disorder (Milich, 2001; Barkley, 2001; Barkley, 1997; Nigg et al., 2002; Lockwood et al., 2001).

A significant correlation of clustering coefficients at certain thresholds was observed between ADHD-I group and controls (for example in the theta band, R=0.82). However, there was not a considerable correlation between ADHD-C and the control group (again in the theta band, R=0.32). The results demonstrated that in ADHDC, there was a rapid reduction at high thresholds while this reduction was much slower in children with ADHD-I and controls. Therefore, in ADHD-C, the functional segregation was significantly reduced at higher thresholds which may be correlated with impairment in the neural network (Clarke et al., 2008; Murias et al., 2006). We suggest that this rapid reduction of theta, alpha, and beta clustering coefficients at higher thresholds may be related to hyperactivity or impulsivity in ADHD-C but not related to the inattention presentation seen in ADHD-I.

Overall, ADHD presentations demonstrated an increased segregation in low thresholds that were correlated to increased local modularity and may be related to inattention. It shows a different functional network in the brains of ADHD-C subjects which was consistent with several studies that introduce ADHD-I as a distinct disorder (Milich, 2001; Barkley, 2001; Barkley, 1997; Nigg et al., 2002). We suggest that decreased clustering at high thresholds may be associated with hyperactivity. Furthermore, in the theta band, significantly increased clustering and decreased global efficiency of the ADHD-C group demonstrates that the small-world brain network has been impaired and a shift toward regular networks was observed.

Our findings are the first to show a significant difference in functional brain networks between children with ADHD-C and ADHD-I. However, further studies with greater number of participants is required to replicate our results and assumptions.
